# Complications after breast augmentation with dermal fillers containing copolyamide: A systematic review^✰^

**DOI:** 10.1016/j.jpra.2024.01.009

**Published:** 2024-01-23

**Authors:** Karin Hedström, Alberto Falk-Delgado, Helena Sackey

**Affiliations:** a Department of Surgery, South General Hospital, Stockholm, Sweden; b Department of Plastic and Craniofacial Surgery, Karolinska University Hospital, Stockholm, Sweden; c Department of Surgical Sciences, Uppsala University, Uppsala, Sweden; d Department of Breast-, Endocrine Tumors and Sarcoma, Karolinska University Hospital, Stockholm, Sweden; e Department of Molecular Medicine and Surgery, Karolinska Institutet, Stockholm, Sweden

**Keywords:** Copolyamide, Dermal filler, Injections, Los Deline, Complications, Breast augmentation

## Abstract

**Background:**

Dermal fillers containing copolyamide are used for breast augmentation and are marketed under different labels, such as Aquafilling, Los Deline, Aqualift, and Activegel. In recent years, the number of publications reporting complications after use of these fillers has increased.

**Methods:**

Through a computerized search following the Preferred Reporting Items for Systematic Reviews and Meta-Analyses guidelines, a systematic review of published studies on complications, treatment options, and radiological findings related to breast augmentation with dermal fillers containing copolyamide was performed. Publications between January 1, 2007, and January 23, 2023, were included. Retrieved studies were screened for inclusion and quality assessment. The Joanna Briggs checklist for case reports and the Strengthening the Reporting of Observational Studies in Epidemiology checklist for cross-sectional studies were used.

**Results:**

Sixteen studies met the inclusion criteria: 14 case reports and 2 retrospective cohort studies, including 196 women and 333 complications. Long-term complications (≥30 days after surgery) were described in 15 studies. The most commonly reported complications were nodules in the breast (130 patients), pain (92 patients), inflammation and/or infection (43 patients), breast deformities (35 patients), and migration of the filler to the pectoralis muscle, abdominal wall, thoracic wall, pubic area, back, or upper extremity (27 patients). The median time between injection of the dermal filler and any complication was 18 months, and the majority of patients with complications required surgical intervention.

**Conclusion:**

Given the reports of severe complications months to years after injection of dermal fillers containing copolyamide and the lack of studies evaluating long-term safety, our interpretation is that dermal fillers containing copolyamide should not be used for breast augmentation.

## Introduction

Breast augmentation using implants is one of the most frequently performed aesthetic surgical procedures worldwide.^1^ It is considered a safe procedure but requires general anesthesia, and recovery can take time. The global aesthetic industry has been searching for less invasive procedures to increase breast volume. Injections with different substances, so-called dermal fillers, are one example. Over the years, different materials have been used for this purpose, including paraffin, various oils, liquid silicon, and collagen, all with different degrees of complications.^2^

During the 1980s, a dermal filler containing polyacrylamide gel (PAAG) was introduced in Ukraine, which was later reported to have high risk of complications.^3^^,^^4^ Since 2006, the use of PAAG has been prohibited in many countries.^5^

More recently, dermal fillers containing copolyamide have been marketed and are used in the medical aesthetic market worldwide. One of these, marketed as Aquafilling, is a dermal filler initially used for facial contouring and has been used in Europe for breast augmentation since 2008.^6^^,^^7^ This dermal filler is a hydrophilic gel composed of 98% sodium chloride solution (0.9%) and 2% copolyamide.^8^, ^9^, ^10^ In 2018, the label of the product changed to Los Deline (BioTrh, s.r.o., Czech Republic).^9^ Aqualift is yet another dermal filler on the market containing copolyamide. In 2015, it was renamed Activegel (National Medical Technologies Center Co., Ltd., Ukraine).^9^

In recent years, the number of publications reporting complications after use of dermal fillers containing copolyamide has increased. These complications include nodules in the breast, breast deformities, inflammation/infection, and migration of the dermal filler, locally into the breast glandular tissue and the pectoralis muscle but also distant migration to the abdominal wall, pubic area, back, and upper extremity.^7^^,^^9^^,^^11^^,^^12^

Additionally, concerns have been raised regarding diagnostic challenges after injection with dermal fillers containing copolyamide, i.e., that the filler may be difficult to distinguish from a malignant lesion^6^^,^^13^ or may hamper breast cancer detection on mammography.^7^^,^^9^^,^^10^

In some countries, such as Korea, Poland, and Italy, there are already bans or strong recommendations not to use this filler for breast augmentation.^14^, ^15^, ^16^

This study aimed to perform a systematic review of complications and imaging findings after breast augmentation with dermal fillers containing copolyamide and assess potential treatment options for these complications.

## Methods

The study was registered at PROSPERO, with the assigned identifying number “CRD42022320649.”

### Search strategy

The study followed the Preferred Reporting Items for Systematic Reviews and Meta-Analyses (PRISMA) guidelines, and the completed checklist is shown in Addendum Table 1. Most studies in this systematic review were case reports. Thus, many of the items in the PRISMA Checklist 2020 were not applicable. We performed a literature search on PubMed (National Library of Medicine), Web of Science (Thomson Reuters), and Embase (Elsevier) for studies with keywords (“mammaplasty” OR “breast enlargement” OR “breast enhancement” OR “breast augmentation” OR (“cosmetic techniques” AND breast) AND (“dermal filler” OR “dermal fillers” OR injections OR injection OR “intradermal injections” OR filler OR fillers OR “filler injection” OR “filler injections” OR “gel injection” OR “gel injections” OR “los deline” OR aquafilling OR “hydrophilic gel” OR copolyamide OR aqualift OR activegel) and covered publication dates from January 1, 2007, to January 23, 2023.

**Table 1:** Summary of all included studies.

Reference	Country	Study design	Study population (No.)	Age, years (range)	Follow-up time, months (range)	Event year(s)	Injected amount of filler in each breast, ml	Method of injection
Arslan,^13^	Turkey	Case report	1	35	—	–	—	US* not used
Basara Akin,^17^	Turkey	Case report	1	37	—	–	—	US not used
Chalcarz,^16^	Poland	Case report	4	—	—	–	100–260	—
Elibol,^18^	Turkey	Case report	1	40	—	–	—	—
Gierej,^12^	Poland	Case report	1	35	1, 6, 10	2020	150	—
Hee Ko,^11^	South Korea	Case report	1	32	—	—	—	—
Huβ,^19^	Germany	Case report	1	39	0.75	—	—	—
Ikizceli,^6^	Turkey	Case report	1	24	—	—	—	—
Jung,^10^	South Korea	Case report + literature review	1	32	24	—	200–250	—
Kim,^20^	South Korea	Case report + literature review	1	49	6	—	—	—
Loesch,^21^	Switzerland	Case report- literature review	1	33	1, 1.5, 12	2020	—	—
Namgoong,^22^⁎⁎	South Korea	Retrospective cohort	146	Mean 34 (26–50)	Mean 10.5 (6–18)	2015–2019	50–300	—
Nomoto,^9^	Japan	Retrospective cohort	29	Mean 42 (26–61)	—	2018–2020	20–250	—
Ozcan,^23^	Turkey	Case report	2	28 and 32	—	—	—	—
Shin,^8^	South Korea	Case report	2	—	6	—	65–95	US not used
Son,^7^	South Korea	Case report	3	32, 42, and 44	15 (only reported for one patient)	—	—	—

If an article neither commented nor negated a specific finding, it was reported as “—.”.

⁎ US=ultrasound.

⁎⁎ In one of the retrospective cohort studies (Namgoong et al.), six patients had buttock augmentation with Aquafilling, and these cases were included in the mean values for age and follow-up.

In addition, a manual web search was performed to find potential studies of interest not published in the databases and websites above. There were no language restrictions. Relevant studies were searched from the date of inception. This systematic review included only studies on breast augmentation with dermal fillers containing copolyamide (i.e., Aquafilling, Los Deline, Aqualift, and Activegel). Studies on other types of dermal fillers not containing copolyamide and studies reporting complications solely after injection in areas other than the breast were excluded.

To find potentially relevant studies, the obtained titles and abstracts were read independently by 2 authors (HS and KH). Included studies were case reports and cohort studies. Letters to the Editor, invited discussions, position statements, and articles exclusively describing an injection method were excluded. The selected studies were read as full text by 2 authors (HS and KH) for final assessment regarding inclusion. If there were dissents, a third author (AFD) was consulted to reach consensus.

### Data extraction

From the included studies, the following variables were obtained independently from 2 authors (HS and KH) and described in the tables: publication year, country, study design, study population (number of patients with complications), mean/median age, follow-up time (months), event year, amount of filler being injected (ml), method of injection, short- and long-term complications, time between injection and complication, nodules, breast deformities, pain, infection, inflammation, migration, fistulas, complications when breastfeeding, neoplasia/atypia, imaging findings, and treatment of complications. If a study neither commented nor negated a specific finding, it was reported as “—” in the tables.

### Quality assessment

To assess the quality of the included studies, the Joanna Briggs checklist (risk of bias tool) for case reports^17^ and the Strengthening the Reporting of Observational Studies in Epidemiology (STROBE) checklist for cross-sectional studies and retrospective cohort studies were used.^18^ The Joanna Briggs checklist included 8 items and the STROBE checklist included 22 items, both with the response options “yes” or “no,” and are shown in Addendum Tables 2 and 3, respectively. When a question was not applicable for the study, it was reported as “—” in the tables.

**Table 2:** Patients with reported long-term complications (≥ 30 days).

Reference	Nodulus	Breast deformities	Pain	Infection	Inflammation	Migration	Fistula	Complications when breastfeeding	Neoplasia/atypia	Time between injection and complication(-s), months (range)
Arslan,^13^	—	1	1	—	1	No	—	—	—	8
Basara Akin,^17^	1	—	1	—	—	—	—	—	—	6
Chalcarz,^16^	—	2	2	—	3	1	—	—	—	1, 3, 20
Elibol,^18^	1	No	No	No	No	No	—	—	—	24
Gierej,^12^	—	1	1	No	1	1	—	—	—	31
Hee Ko,^11^	—	1	1	1	1	1	—	—	—	6
Huβ,^19^	—	1	1	1	1	1	—	—	No	—
Ikizceli,^6^	—	—	1*	—	—	—	—	—	—	—
Jung,^10^	No	No	1	1	1	No	1	—	—	36
Kim,^20^	No	1	1	No	No	No	—	—	—	12
Loesch,^21^	—	1	1	1	1	No	—	1	—	60
Namgoong,^22^	122	9	76	1	10	12⁎⁎	—	1	1	39 (5–144)
Nomoto,^9^	5	17	3	8	8	4⁎⁎⁎	—	—	—	22 (1–48)
Ozcan,^23^	No	1	1	—	—	No	—	—	—	12 and 48
Shin,^8^	No	No	No	No	No	No	—	No	No	—
Son,^7^	1	—	1	1	1	3	2	1	—	4, 11, and 12

∗ Did not mention when the complication occurred.

∗∗ Not described if it was distant migration or migration into the breast or pectoralis muscles.

⁎⁎⁎ One patient with migration beneath the pectoralis muscle.

**Table 3:** Findings on imaging.

Reference	Nodules (solid lesions)	Appearance in pectoralis muscle	Distant migration	Edema	Calcifications	Findings on mammography	Findings on US	Findings on MRI	Findings on CT
Arslan,^13^	Yes (1 patient)	Yes (1 patient)	No	Yes (1 patient)	—	—	Yes	Yes	—
Basara Akin,^17^	Yes (1 patient)	No	No	No	No	Yes	Yes	Yes	—
Chalcarz,^16^	—	—	No	—	—	—	Yes	Yes	—
Elibol,^18^	No	No	No	No	No	Yes	Yes	Yes	—
Gierej,^12^	No	—	Yes (1 patient)	No	No	—	Yes	Yes	Yes
Hee Ko,^11^	Yes (1 patient)	No	Yes (1 patient)	No	No	—	Yes	Yes	Yes
Huβ,^19^	Yes (1 patient)	No	Yes (1 patient)	No	No	—	Yes	—	—
Ikizceli,^6^	Yes (1 patient)	No	No	No	No	Yes	Yes	Yes	—
Jung,^10^	No	Yes (1 patient)	No	No	No	—	Yes	—	Yes
Kim,^20^	No	Yes (1 patient)	No	No	No	—	Yes	Yes	—
Loesch,^21^	No	No	No	No	—	—	Yes	—	—
Namgoong,^22^	—	—	—	—	Yes (1 patient)	—	Yes	Yes	Yes
Nomoto,^9^	—	Yes (1 patient)	Yes (1 patient)	No	—	—	—	Yes	Yes
Ozcan,^23^	Yes (2 patients)	Yes (1 patient)	No	No	No	—	Yes	Yes	—
Shin,^8^	—	—	—	—	—	—	—	—	—
Son,^7^	—	Yes (1 patient)	Yes (1 patient)	—	—	Yes	Yes	Yes	Yes

## Results

### Included studies

**Figure 1:**
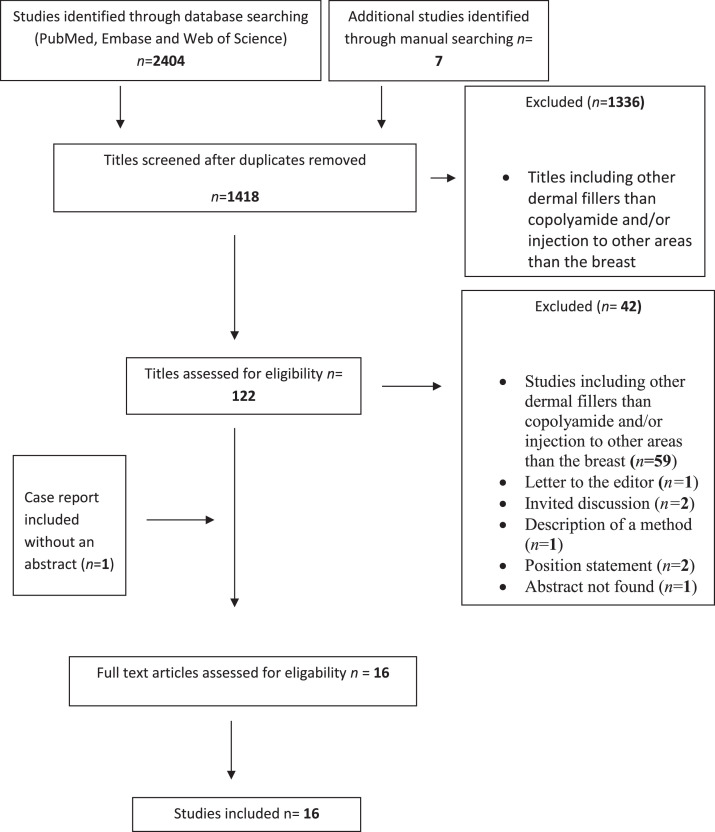
PRISMA flowchart of the identification and inclusion of studies in the systematic review.

The search strategy resulted in 1418 publications after removal of duplicates. Sixteen studies met the inclusion criteria: 14 case reports (including up to 4 patients) and 2 retrospective cohort studies. Three studies were found on ResearchGate, a database indexed by Google Scholar by manually searching the web. The PRISMA flowchart of identification and inclusion of the studies in this systematic review is presented in Figure 1.

Fifteen of the 16 included studies reported complications,^6^^,^^7^^,^^9^, ^10^, ^11^, ^12^, ^13^^,^^19^, ^20^, ^21^, ^22^, ^23^, ^24^, ^25^, ^26^ and one study solely described a method of using Aquafilling to fill deformities after breast augmentation with implants.^8^

All in all, 196 women who had injections with dermal fillers containing copolyamide were included in this systematic review. The majority (*n* = 191) had injections with Aquafilling/Los Deline,^6^, ^7^, ^8^^,^^10^^,^^11^^,^^13^^,^^19^, ^20^, ^21^, ^22^, ^23^, ^24^, ^25^, ^26^ and one study reported 5 women who had injections with Aqualift/Activegel^9^ (Table 1).

### Risk of bias

None of the 14 case reports^6^, ^7^, ^8^^,^^10^^,^^11^^,^^13^^,^^19^, ^20^, ^21^, ^22^, ^23^, ^24^^,^^26^ fulfilled the criteria for low risk of bias according to the Joanna Briggs checklist for case reports. We converted the response option “yes” in the checklist to one point and added up the answers. Eleven of the 14 case reports had more than half of the maximum points (Addendum Table 2).

None of the 2 retrospective cohort studies^9^^,^^25^ fulfilled the criteria for low risk of bias according to the STROBE checklist for cross-sectional studies. Of the 22 items on the checklist, 11 and 10 were described in the 2 retrospective cohort studies, respectively (Addendum Table 3).

### Demographics

The included studies originated from South Korea (*n* = 6), Turkey (*n* = 5), Poland (*n* = 2), Germany (*n* = 1), Switzerland (*n* = 1), and Japan (*n* = 1) and are presented in Table 1. The ages of the included patients ranged from 24 to 61 years but were not reported in 2 of the case reports. The median age of the participants was 36 years in the remaining 12 case reports and 34 and 42 years in the 2 retrospective cohort studies, respectively.

### Complications

**Figure 2:**
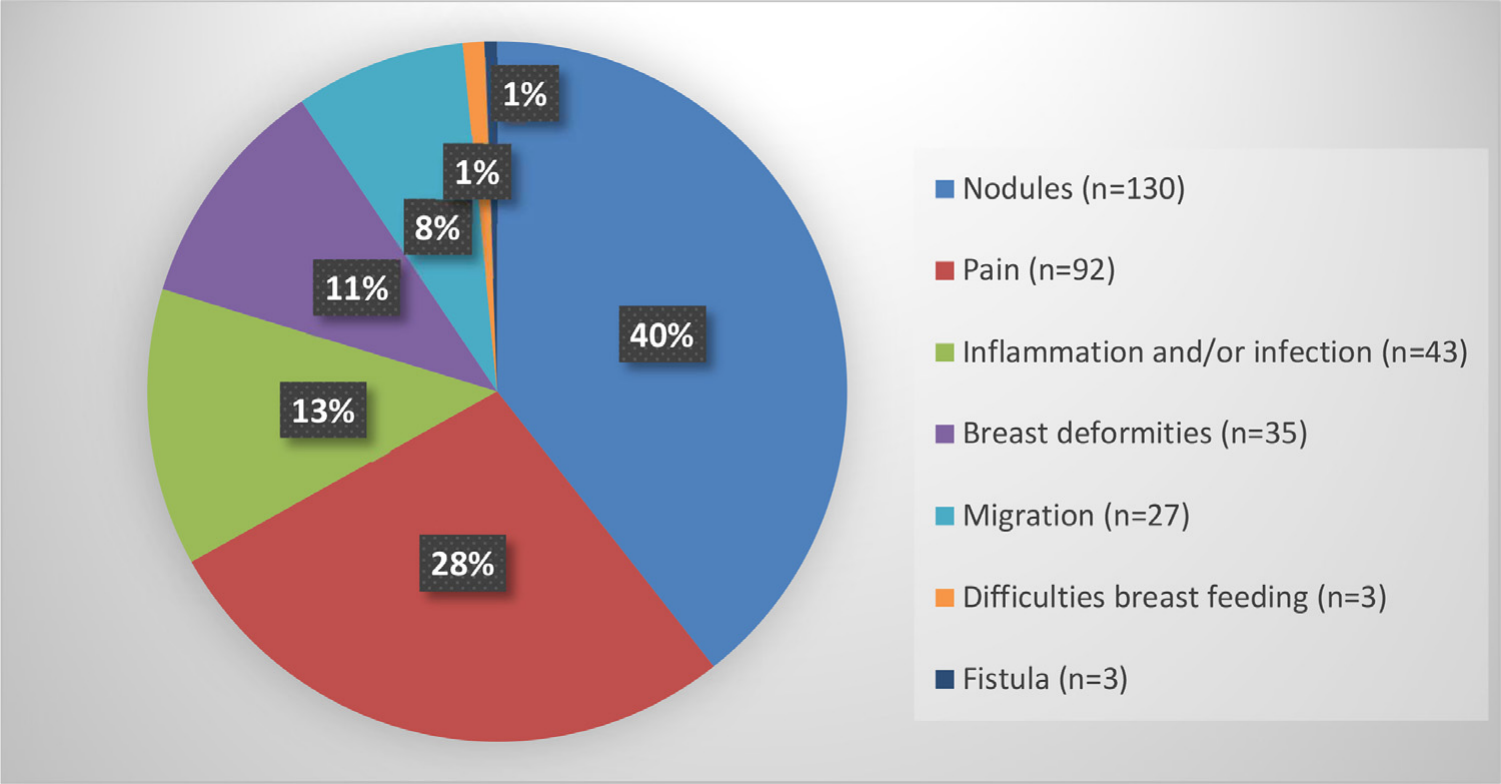
Reported long-term complications. Percentages may not add up to 100% owing to rounding.

In total, 196 women and 333 complications were reported in the included studies (Figure 2). Complications were divided into short-term (occurred <30 days after injection of the dermal filler) and long-term (occurred ≥30 days after injection of the dermal filler).

One study reported a patient who developed infectious symptoms immediately after injecting Activegel into the breast.^9^ No other short-term complications were reported in any of the other studies.

Long-term complications were reported in 15 of the 16 included studies and occurred 1 to 60 months after dermal filler injection, with a median of 18 months (Table 2). The most commonly reported long-term complications were nodules in the breast (*n* = 130),^7^^,^^9^^,^^20^^,^^21^^,^^25^ followed by pain (*n* = 92),^6^^,^^7^^,^^9^, ^10^, ^11^, ^12^, ^13^^,^^19^^,^^20^^,^^22^, ^23^, ^24^, ^25^, ^26^ inflammation and/or infection in the breast (*n* = 43),^7^^,^^9^, ^10^, ^11^, ^12^, ^13^^,^^19^^,^^22^^,^^24^^,^^25^ breast deformities presenting as volume loss and/or swelling (*n* = 35),^9^^,^^11^, ^12^, ^13^^,^^19^^,^^22^^,^^24^, ^25^, ^26^, ^27^ and distant migration of the filler (*n* = 23).^7^^,^^9^^,^^11^, ^12^^,^^19^^,^^22^^,^^25^ Three patients had difficulties when breastfeeding due to pain, galactocele, and/or mastitis,^7^^,^^24^^,^^25^ and one patient had sepsis.^25^

Distant migration was reported in 23 patients: to the abdominal wall (*n* = 5),^7^^,^^9^^,^^11^^,^^19^^,^^22^ pubic area (*n* = 3),^7^^,^^9^^,^^11^ thoracic wall (*n* = 2),^7^ back (*n* = 1),^9^ and upper extremity (*n* = 1).^12^ Further, 12 patients in one of the retrospective cohort studies were reported to have migration of the dermal filler without a description of the location.^25^

Fistulas where the filler material is secreted from an opening in the skin were reported in 3 patients: 2 patients had a fistula in the breast,^7^^,^^10^ and 1 patient with distant migration had a fistula in the pubic area.^7^

### Imaging findings

The presence of the dermal filler in locations other than the avascular plane between the pectoralis muscle and the breast glandular tissue was described in 13 patients in 10 different case reports and 16 patients in the 2 retrospective cohort studies^7^^,^^9^, ^10^, ^11^, ^12^, ^13^^,^^19^^,^^22^^,^^23^^,^^25^^,^^26^ (Table 3). In 9 patients, there was visible filler material in the breast glandular tissue and/or the subcutaneous fat,^6^^,^^7^^,^^10^^,^^11^^,^^13^^,^^26^ 6 patients in the pectoralis muscle,^7^^,^^9^^,^^10^^,^^13^^,^^23^^,^^26^ and 8 patients in more distant locations such as the abdominal wall, pubic area, thoracic wall, back, and upper extremity.^7^^,^^9^^,^^11^^,^^12^^,^^19^^,^^22^ These findings were detected using magnetic resonance imaging (MRI) in 6 patients,^7^^,^^12^^,^^13^^,^^23^^,^^26^ ultrasound in 5 patients,^7^^,^^10^^,^^12^^,^^13^^,^^23^ and computed tomography in 4 patients.^7^^,^^9^, ^10^, ^11^

Nodules in the breast were described in 7 patients with different imaging modalities: mammography,^6^^,^^20^ ultrasound,^11^^,^^13^^,^^20^^,^^22^^,^^26^ and MRI.^6^^,^^20^^,^^26^

In one case report, the authors illustrated the difficulty distinguishing between filler material in the breast and so-called mucocele-like lesions, a rare benign lesion with highly variable upgrade rates after excision.^20^

Six patients in 4 case reports underwent mammography.^6^^,^^7^^,^^20^^,^^21^ The authors of these studies described that the breast tissue appears very dense on mammography after dermal filler injections and recommended additional imaging modalities, including ultrasound and/or MRI, for better diagnostics.

### Treatment of complications

**Figure 3:**
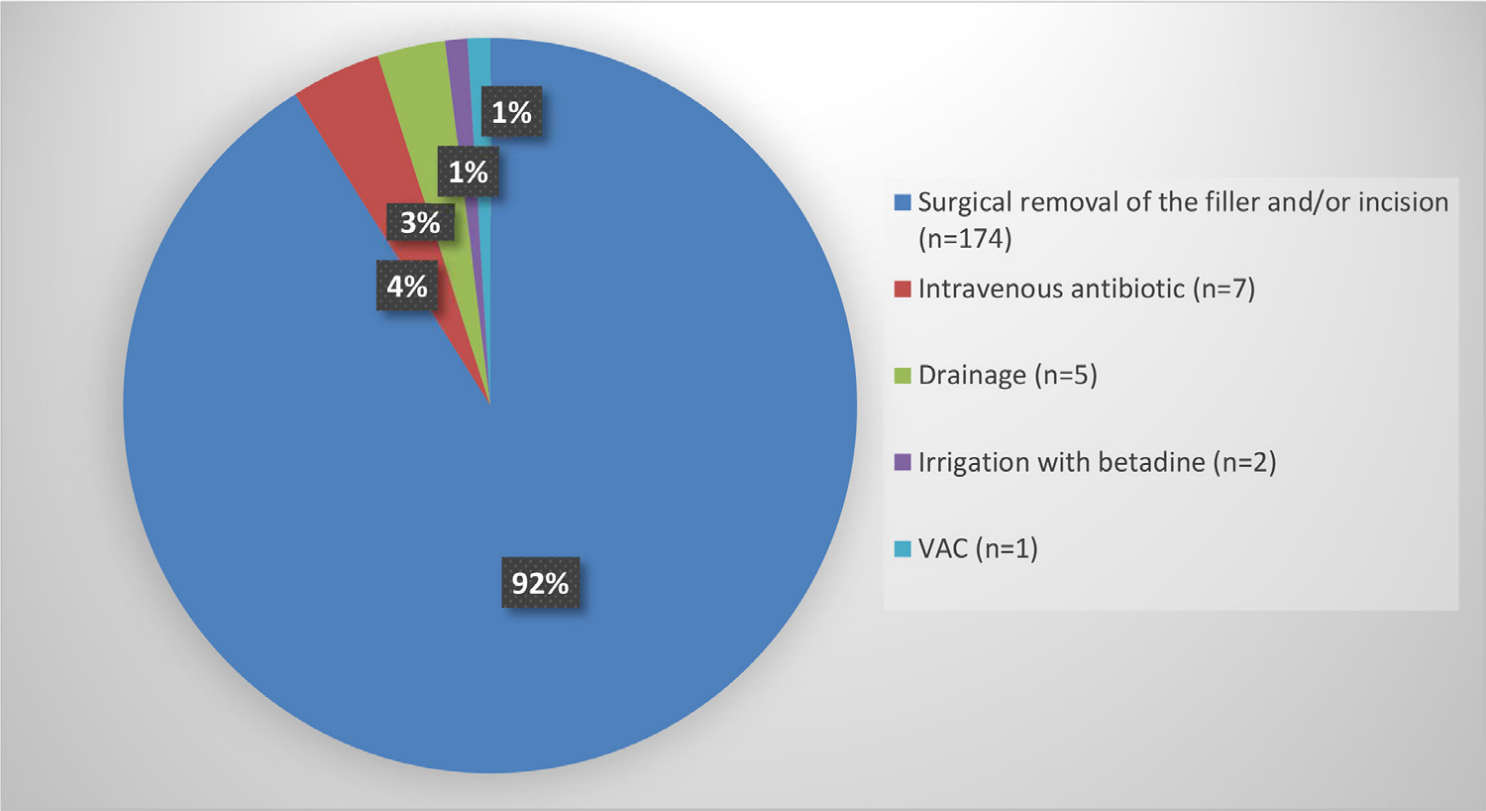
Treatment of complications. Percentages may not add up to 100% owing to rounding. Patients may have more than one treatment.

Treatment of complications is presented in Table 4 and Figure 3. The majority of patients with complications underwent surgical removal of the dermal filler and/or an incision in the breast (*n* = 174).^7^^,^^9^, ^10^, ^11^, ^12^, ^13^^,^^19^^,^^22^, ^23^, ^24^, ^25^ In addition, 7 patients were treated with intravenous antibiotics, 5 patients with drainage, 2 patients had irrigation with betadine, and 1 patient had vacuum-assisted closure.^10^, ^11^, ^12^^,^^23^, ^24^, ^25^

**Table 4:** Treatment of complications.

Reference	Drainage	Incision	Surgical removal of the filler	Mastectomy	Antibiotics (po/iv/topic)	Other local treatment
Arslan,^13^	No	No	Yes (1 patient)	No	Yes (1 patient)	No
Basara Akin,^17^	No	No	No	No	No	No
Chalcarz,^16^	No	Yes (3 patients)	Yes (1 patient)	No	No	No
Elibol,^18^	No	No	No	No	No	No
Gierej,^12^	Yes (1 patient)	Yes (1 patient)	Yes (1 patient)	No	No	No
Hee Ko,^11^	Yes (1 patient)	Yes (1 patient)	No	No	Yes (1 patient)	No
Huβ,^19^	No	Yes (1 patient)	No	No	Yes (1 patient)	No
Ikizceli,^6^	No	No	No	No	No	No
Jung,^10^	Yes (1 patient)	Yes (1 patient)	Yes (1 patient)	No	Yes (1 patient)	Yes (1 patient)
Kim,^20^	Yes (1 patient)	No	Yes (1 patient)	No	Yes (1 patient)	Yes (1 patient)
Loesch,^21^	No	No	Yes (1 patient)	No	Yes (1 patient)	Yes (1 patient)
Namgoong,^22^	No	No	Yes [146 patients (6 gluteal)]	Yes (1 patient)	Yes (2 patients)	No
Nomoto,^9^	—	—	Yes (13 patients()	—	—	—
Ozcan,^23^	No	No	No	No	No	No
Shin,^8^	No	No	No	No	No	No
Son,^7^	Yes (1 patient)	Yes (1 patient)	Yes (1 patient)	No	Yes (1 patient)	No

A patient can have several treatments for complications.

∗ Not specified if surgical removal or only incision.

One case report presented a patient who was admitted due to pain 5 months after the excision of atypical ductal hyperplasia (ADH) in the breast. This patient had an injection with Los Deline 36 months before the excision of the ADH, and an ultrasound revealed injected filler material in the retroglandular area. The patient underwent bilateral mastectomy with immediate breast reconstruction; however, the indication for mastectomy is not thoroughly described in the study.^25^

## Discussion

This is the first systematic review of complications after breast augmentation with dermal fillers containing copolyamide, including 16 studies with 196 women and 333 complications. Long-term complications were described in 15 studies, and the most commonly reported were nodules in the breast,^7^^,^^9^^,^^20^^,^^21^^,^^25^ pain,^6^^,^^7^^,^^9^, ^10^, ^11^^,^^13^^,^^19^^,^^20^^,^^22^, ^23^, ^24^, ^25^, ^26^ inflammation and/or infection,^7^^,^^9^, ^10^, ^11^^,^^13^^,^^19^^,^^22^^,^^24^^,^^25^ breast deformities,^9^^,^^11^^,^^13^^,^^19^^,^^22^^,^^24^, ^25^, ^26^ migration of the filler,^7^^,^^9^^,^^11^^,^^12^^,^^19^^,^^22^^,^^25^ problems when breastfeeding,^7^^,^^24^^,^^25^ and fistulas.^7^^,^^10^ For all but one patient, complications occurred later than 1 month after injection of the dermal filler, with a median of 18 months. The majority of women with complications (92%) needed surgical removal of the filler and/or an incision in the breast.

Although the reported complications may be severe and often require surgical intervention, no published studies evaluated the safety of injection with dermal fillers containing copolyimide. Thus, the incidence of complications is unknown.

One of the more severe complications is migration of the dermal filler, which was reported in 12 of the 16 included studies in this systematic review.^6^^,^^7^^,^^9^, ^10^, ^11^, ^12^, ^13^^,^^19^^,^^22^^,^^23^^,^^25^^,^^26^ The migration was local, into the breast glandular tissue, subcutaneous fat, and the pectoralis muscle; however, distant migration to the pubic area, abdominal wall, thoracic wall, back, upper extremity, and hand was also reported. It is unclear if the described migration into the breast glandular tissue and the pectoralis muscle was migration per se or if the dermal filler was injected into these areas. When injecting a dermal filler for breast augmentation, one should aim for the avascular plane between the breast glandular tissue and the pectoralis muscle. The manufacturer of Aquafilling/Los Deline recommends ultrasound for this procedure. However, in this systematic review, none of the studies described ultrasound-assisted injections of the filler. Our own experience after treating patients with complications after injection with dermal fillers containing copolyamide is that the filler material is diffusely spread in the breast glandular tissue, subcutaneous fat, and the pectoralis muscle, making it hard to separate from normal breast parenchyma. Repeated surgical procedures may be required to remove all filler material.

The reported complications after injection with dermal fillers containing copolyamide are mainly the same type of complications as described for dermal fillers containing PAAG, a filler prohibited in many countries due to serious complications when used for breast augmentation.^4^^,^^27^ As stated, dermal fillers containing copolyamide have been used for breast augmentation in Europe since 2008 and are marketed under different labels, such as Aquafilling, Los Deline, Aqualift and Activegel.^8^, ^9^, ^10^ These dermal fillers are hydrophilic gels composed of 98% sodium chloride solution (0.9%) and 2% copolyamide, a polymer with amide bonds containing aromatic rings.^8^, ^9^, ^10^ In a study from Japan, nuclear magnetic resonance spectroscopy was used to compare the composition of copolyamide and PAAG.^9^ The conclusion was that the 2 dermal fillers closely resemble each other and cause the same type of complications. Thus, the authors advised against further use of fillers containing copolyamide until long-time safety has been established.

In addition to reported complications, a few authors raised concerns regarding diagnostic difficulties after injection with dermal fillers containing copolyamide. Six patients in 4 case reports had very dense breast tissue on mammography after injection with dermal fillers containing copolyamide. According to the authors, this appearance could hamper the full evaluation of the breast parenchyma; hence, they recommended additional imaging modalities, including ultrasound and/or MRI.^6^^,^^7^^,^^20^^,^^21^ Even so, regarding breast imaging after breast augmentation with dermal fillers containing copolyamide, no published studies compared different imaging modalities in regard to detecting the dermal filler or evaluating the risk that the filler may obstruct the detection of concomitant cancer.

Another concern raised by some authors is that patients may develop calcifications after injection of dermal fillers in the breast, calcifications that may be difficult to distinguish from malignant lesions.^6^ However, none of the included studies reported a patient who developed calcifications or that the dermal filler masked a cancer. Therefore, the number of patients in the included studies is too few, and the follow-up time is too short to draw conclusions regarding this.

Limitations in this systematic review are related to the lack of prospective studies on the topic, and there are no published studies evaluating the incidence of complications after injection with dermal fillers containing copolyamide. The included studies were single-center case series with a limited number of patients and 2 cross-sectional studies, and none fulfilled the criteria for low risk of bias according to the Joanna Briggs and STROBE checklists.

The strengths included the prospectively registered protocol for this systematic review, a broad search strategy, no language restrictions, and an independent parallel assessment of all identified studies by 2 experienced surgeons. The systematic review followed the PRISMA criteria. To our knowledge, this is the first systematic review on complications after breast augmentation with dermal fillers containing copolyamide, which was conducted according to best practice.

Many of the authors of the included studies in this systematic review concluded that long-term safety has not yet been evaluated^10^^,^^11^^,^^22^^,^^23^ and suggested that breast augmentation with fillers containing copolyamide should come to an end. The Academic Society of Aesthetic and Reconstructive Breast Surgery of Korea declared that copolyamide has the same composition as PAAG and therefore opposed using fillers containing copolyamide for breast augmentation.^14^ The Polish Society of Plastic, Reconstructive and Aesthetic Surgery banned the use of Los Deline in 2020 after a chemical analysis revealed that the substance was similar to PAAG.^15^ In the United States, all dermal fillers are prohibited for breast augmentation. According to the Food and Drug Administration, fillers can cause permanent side effects if used for large-scale body contouring, i.e., breast or buttock augmentations.^28^ Furthermore, in 2022, the Italian Aesthetic Medicine Association made a position statement and concluded that the entry of Los Deline into the Italian market should be postponed until the safety of the product is evaluated.^16^

In conclusion, a growing body of data indicates complications months to years after injection of dermal fillers containing copolyamide, complications that can be severe and often require surgical intervention. Given the number of reports, the risk of severe complications, and a lack of studies evaluating long-term safety, the interpretation is that dermal fillers containing copolyamide should not be used for breast augmentation.

## Declaration of competing interest

The authors declare no conflict of interest.
